# M. Zola on French Journalism and Its Health Results

**Published:** 1889-05-18

**Authors:** Bowness Fischer


### M. Zola on French Journalism and its Health Results.

By Colonel Bowness Fischer.

In an interesting book which has just been published on Parisian life by M. Charles Chincholle, of the *Figaro*, M. Zola enters a pathetic protest against the hurry of the age in which we live, and the consequence this hurry produces both on the characters of those who write and of those who read the French newspapers of the day. "We are," he says, "more and more overwhelmed under a heap of blackened paper that rolls down upon us every morning. Where do all these old newspapers go to? It is terrible to think of, the numbers of them which disappear, utterly useless, out of date in a couple of hours, and of such inferior paper that they can't even be used to wrap up tallow candles. I think of my grandfather, and of the slow, earnest manner with which *he* settled himself in his armchair to read *his* newspaper. He gave up at least three or four hours to it; not a line was passed over; all filed past him from the title of the newspaper to the signature of the manager at the end of it. This done, he carefully folded it up and arranged it, according to its date, on a shelf, for he kept the whole collection. I have seen, during twenty years, a black closet filling with this collection without anyone thinking of touching a number. No other newspaper entered my grandfather's house; and but one newspaper existed for him—his own. How everything is changed to-day! We open a paper, run through it, and throw it away. I doubt whether there is anyone sufficiently artless to encumber himself with a collection, for we all know that facts have only the interest of their hour. Besides that, it is no longer one newspaper, but four, five, or even more on the morning of a political crisis, that we buy and crumple up when we have read the twenty odd lines that interested us. Then it all goes to the gutter, and the trampled paper, maculated by our fevers of the day, floats down our streets! And so it comes to pass that the first exclamation of every man who can escape from Paris for a change of a few weeks is, 'At last I shall read no more newspapers!' Oh! to know absolutely nothing is, after our debaucheries of information, utter delight, an earthly paradise! To awake every morning in some quiet corner of the world with our ears calm, utterly ignorant of what the eternal puppets of politics might have said or done on the previous evening, and to go to bed every evening without being acquainted with the follies of the day—it is like a really fresh bath and causes a sensation of overflowing health. Never does one better understand the danger of the fever which hurries all of us into that passionate curiosity which the contemporary Press only serves to intensify. I am aware that at the end of two or three days we become weary of the silence around us, and that we rush off to the nearest station to buy a newspaper, but this is but a proof of the depth of the evil. The virus of information at any price has penetrated us to our bones, and we become like those confirmed tipplers who waste away immediately they are deprived of the poison which is killing them. It would be delightful to have the skull free from all the noise of the Age; but the head of the man of to-day is heavy with the fearful heap of things that journalism daily deposits in it anyhow, in any order, or none. In the country, consequently, we actually envy the ignorant man who passes by—even the peasant worn out with toil, with the vacant eyes of an old beast of burden! It is the old, old quarrel between ignorance and science. There is a virility, a widening of ourselves in continually knowing more and more. Our modern theory of the citizen who knows his rights and governs himself is undoubtedly one of high human dignity. But, from the point of view of happiness, the result certainly seems doubtful to me. I fancy that the nerves of France were much more calm and that her health was more stable when she analysed herself, with less fever, and when hundreds of newspapers did not bring her every morning a detailed account, too often exaggerated, of her slightest ailments. In what is called the neurosis of the Age, in this increasing over-excitement which is transforming the nation and leading it astray, the journalism of the day plays the principal part. Is it not this which exasperates and propagates agitation? We find, therefore, that every autocratic government begins by muzzling the Press, for there is no better way of calming public opinion. Immediately they do so the heads of everyone begin to cool, their bodies grow fat, and a period of material prosperity sets in. The nation, in fact, is at grass—no longer thinking, but quietly browsing. I am far from saying that this fatted sleep is not likely to be followed by a terrible awakening; but it is none the less true that the human animal seems to require these full-bellied slumbers in the cool of the meadows, and to need these hours of utter animal life, in which the only sensation is one of pleasure in being alive. See what we have come to after eighteen years of parliamentary palaver and a free Press! What disgust there is with politics! How wearying it is to govern ourselves! And how enervating it is to acknowledge our illness, and to watch it getting worse minute by minute! If our Assemblies are unpopular, it is because our attention is too much attracted to them, and that they make too much noise for the little work they manage to do. And if we should throw ourselves to-morrow into the arms of a master, it would only be from an ardent desire to go to bed at last, to blow out the candle, and to be able to sleep as long as we liked amidst the profound silence of our street."

So far M. Zola; and it is instructive to find a man who was so potent a factor in the creation of French *Actualité*, not only raising his voice against his own Frankenstein, but invoking the powers of coercion to suppress it. But this love of some new thing is by no means common only to our French friends; it is inherent in human nature; may be found among the Athenians of St. Paul's day, and traced back to the time of Koheleth, or even further still. Deducting its Zolaism, the fable may be narrated of ourselves; and it is evident that while the great world has been spinning "down the ringing grooves of change," this initial inquisitiveness has been increasing in velocity until we see what we now see. It is possible that this is due in part to what de Musset called "le mal du siècle:"

> For we, brought forth and reared in hours
> Of change, alarm, surprise—
> What shelter to grow ripe is ours?
> What leisure to grow wise?

But there is little doubt that it is also due to the "pace" at which we live; and that, unless we are within the shadow of the coming race, when, as Theophrastus Such says, "The creatures who are to transcend and finally supersede us will be steely organisms giving out the effluvia of the laboratory, and performing with infallible exactness more than everything that we have performed with a slovenly approximateness and self-defeating inaccuracy," we must either moderate this pace, or deteriorate mentally, morally, and physically. "Very few, even of stupid men," says a vigorous writer in a recent number of the *Hospital*, "would affirm that full gallop is the right pace for everyday work," and he thinks that if a standard of thought power could be applied "to test the thousands of public writers by it, and to demonstrate how many of them were capable of original thought and of persistent independent action, the proportion would be painfully small." The moral of it, therefore, seems to be that—without going back to M. Zola's grandfather, or to our own admirable ancestor, "Old Leisure," who, George Eliot tells us, "was a contemplative, rather stout gentleman, of excellent digestion, and of quiet perceptions undiseased by hypothesis"—it is absolutely incumbent upon us to change the bad habits that have grown upon us with as little delay as possible. But how this change is to be effected is "another pair of sleeves." And what journalist is first to begin the change is a question as fraught with consequence as the equally important question which nation is to set an example towards "the general disarmament" which the peace-makers promise Europe.

